# Supplementary material for: SpoVG Is Necessary for Sporulation in Bacillus anthracis
Source: Microorganisms. 2020 Apr 10;8(4):548. doi: 10.3390/microorganisms8040548 (PMC7232415; doi:10.3390/microorganisms8040548)
Supplement: Supplementary file 1 [file microorganisms-08-00548-s001.pdf]

Table S1. Primer sequences.

oligonucleotides	sequence (5′-3′) [a]	note
spoVG-up-F	TTTGGTCTCATGACGGATTGTTGAGGCTGAGTAA	amplification of *spoVG* up fragment
spoVG-up-R	TTTGGTCTCACTCC CTTGTGTTCACCACCCTTT	
spoVG-down-F	TTTGGTCTCAATCGGGTTGAGTTTGAAGAAGCG	amplification of *spoVG* down fragment
spoVG-down-R	TTTGGTCTCACCAACATGACCATTCTCATTACGAAC	
spoVG-US-F	TCAGGTTCTTCTAAGCGAAT	screening for *spoVG* deletion strains
spoVG-US-R	TGTCAATGGTTCAGATACGA	
spoVG-SD-F	TGGAGAATGGTTACAAGAGC	
spoVG-SD-R	AGCCGATACAATATGACCTTC	
spoVG-in-F	GAAGTGACTGACGTAAGATTAC	
spoVG-in-R	ACCTCTTCTAACTCGCCTAA	
R*spoVG*-F	CGGGATCCATGGAAGTGACTGACGTAAG	amplification of *spoVG* ORF fragment
R*spoVG*-R	CCCAAGCTTTTACGAAGCGCCCGCTTCTT	
spoIIB-F	ACGCGTCGACATTGATTTTCTTTCTTCCC	amplification of *spoIIB* fragment containing P_{spoIIB}
spoIIB-R	GCTCTAGATTTTACGACGGCTAACAGCT	
spoIIE-lacZ-F	ACGACGGCCAGTGCCAAGCTTGTTCATACCCGTGAGGTC	amplification of P_{spoIIE} fragment for LacZ assay
spoIIE-LacZ-R	ATCAATTCAAGCTGGGGATCCCATTCGCAACTTACTCGT	

pHT304-LacZ-F	CGCCAGGGTTTTCCCAGTCACGAC	verification of pHT304-P$_{spoIIE}$-LacZ
pHT304-LacZ-R	GCGCTCAGGTCAAATTCAGACGGC	
RT-qPCR-*spoIIM*-F	TGTAATTGGAGCGGTGTT	Primer used to RT-qPCR analysis
RT-qPCR-*spoIIM*-R	AATAGCCTCAACGACTTCTT	
RT-qPCR-*spoIIQ*-F	TGACATTGCTGCGAAGAA	
RT-qPCR-*spoIIQ*-R	ACAACATAACCAAGAAGTGAATC	
RT-qPCR-*cotE*-F	GAATAATGAGCCAACAAG	
RT-qPCR-*cotE*-R	ACGATAGCCAATACTTAC	
Q*gatB*_Yqey_F	CGGCATAACAGCAGTCATCA	internal reference of RT-qPCR
Q*gatB*_Yqey_R	AGCTGGTCGTGAAGACCTTG	

[a] Restriction enzyme sites are underscored

Table S2. List of all differentially-expressed proteins identified via iTRAQ analysis

Accession	Description	Abundance Ratio: ΔspoVG /A16R
BA_0314	BA_0314 ABC transporter substrate-binding protein	2.076
BA_1326	BA_1326 maoC family protein	2.011
BA_1450	BA_1450 proton/glutamate symporter family protein	1.972
BA_0093	sigH RNA polymerase factor sigma-70	1.9
BA_3642	BA_3642 oligopeptide ABC transporter substrate-binding protein	1.745
BA_5529	murA1 UDP-N-acetylglucosamine 1-carboxyvinyltransferase	1.716
BA_4807	BA_4807 HD domain-containing protein	1.693
BA_1564	panD aspartate alpha-decarboxylase	1.669
BA_2775	acoB TPP-dependent acetoin dehydrogenase E1 beta-subunit	1.627
BA_4359	proC-3 pyrroline-5-carboxylate reductase	1.626
BA_2754	BA_2754 TetR family transcriptional regulator	1.614
BA_0510	pflA pyruvate formate-lyase-activating enzyme	1.612
BA_4255	mtnW 2,3-diketo-5-methylthiopentyl-1-phosphate enolase	1.608
BA_0728	BA_0728 ABC transporter substrate-binding protein	1.576
BA_5668	BA_5668 major facilitator family transporter	1.567
BA_5626	BA_5626 4-oxalocrotonate tautomerase	1.556
BA_4252	mtnK methylthioribose kinase	1.529
BA_4481	metC-2 cystathionine beta-lyase	1.526
BA_1981	BA_1981 siderophore biosynthesis protein	1.523
BA_4293	BA_4293 sodium-dependent symporter family protein	1.52
BA_0351	BA_0351 iron compound ABC transporter substrate-binding protein	1.491
BA_0509	pfl formate acetyltransferase	1.482
BA_1292	sinR transcriptional regulator SinR	1.48
BA_4035	divIVA cell-division initiation protein DivIVA	1.479
BA_3594	cspB-2 cold shock protein CspB	1.478

BA_5512	wecC UDP-N-acetyl-D-mannosamine dehydrogenase	1.475
BA_4489	BA_4489 5-formyltetrahydrofolate cyclo-ligase	1.468
BA_0352	BA_0352 pyridine nucleotide-disulfide oxidoreductase family protein	1.462
BA_2373	BA_2373 mbtH-like protein	1.46
BA_5424	cspC cold shock protein CspC	1.449
BA_4576	BA_4576 acetyltransferase	1.445
BA_2774	acoC branched-chain alpha-keto acid dehydrogenase subunit E2	1.438
BA_2773	acoL dihydrolipoamide dehydrogenase	1.423
BA_1270	sucA 2-oxoglutarate dehydrogenase E1 component	1.423
BA_4049	murG UDPdiphospho-muramoylpentapeptide beta-N- acetylglucosaminyltransferase	1.415
BA_3988	acpP acyl carrier protein	1.414
BA_3206	BA_3206 M20/M25/M40 family peptidase	1.411
BA_0174	BA_0174 ABC transporter ATP-binding protein	1.41
BA_1240	BA_1240 acetyltransferase	1.386
BA_0348	fucA L-fuculose phosphate aldolase	1.383
BA_1832	BA_1832 acetyltransferase	1.38
BA_4256	mtnX 2-hydroxy-3-keto-5-methylthiopentenyl-1-phosphate phosphatase	1.366
BA_3048	BA_3048 ABC transporter ATP-binding protein	1.361
BA_2371	dhbB isochorismatase	1.352
BA_1194	BA_1194 oligopeptide ABC transporter ATP-binding protein	1.344
BA_5663	thiD-2 pyridoxal kinase	1.341
BA_4480	metC-1 cystathionine gamma-synthase	1.33
BA_3665	BA_3665 glycerol-3-phosphate acyltransferase PlsY	1.319
BA_0121	rplX 50S ribosomal protein L24	1.315
BA_0072	folB dihydroneopterin aldolase	1.313
BA_4827	gap-1 glyceraldehyde-3-phosphate dehydrogenase	1.311
BA_4608	udk uridine kinase	1.31
BA_3690	alkK medium-chain-fatty-acid--CoA ligase	1.307
BA_1383	BA_1383 hypothetical protein	1.303
BA_0499	glsA-1 glutaminase	1.3
BA_2776	acoA TPP-dependent acetoin dehydrogenase E1	1.296

	alpha-subunit	
BA_2899	BA_2899 aspartate aminotransferase	1.293
BA_5422	yfiA ribosomal subunit interface protein	1.292
BA_1421	leuB 3-isopropylmalate dehydrogenase	1.287
BA_5581	spo0F stage 0 sporulation protein F	1.285
BA_4804	pheS phenylalanyl-tRNA synthetase subunit alpha	1.282
BA_5043	BA_5043 mutT/nudix family protein	1.277
BA_2691	BA_2691 endoribonuclease L-PSP	1.274
BA_0729	tenI transcriptional regulator TenI	1.274
BA_0657	BA_0657 oligopeptide ABC transporter permease	1.267
BA_4483	BA_4483 ArsR family transcriptional regulator	1.266
GBAA_pXO1_0155	GBAA_pXO1_0155 resolvase	1.263
BA_4950	trmB tRNA (guanine-N(7)-)-methyltransferase	1.263
BA_0502	BA_0502 penicillin-binding protein	1.259
BA_5584	rpoE DNA-directed RNA polymerase subunit delta	1.257
BA_2370	dhbE 2,3-dihydroxybenzoate-AMP ligase	1.257
BA_0766	BA_0766 nitroreductase	1.253
BA_4258	BA_4258 5-methylthio-3-oxo-1-penten-1,2-diol dioxygenase	1.251
BA_1195	BA_1195 oligopeptide ABC transporter ATP-binding protein	1.251
BA_0312	BA_0312 ABC transporter ATP-binding protein	1.25
BA_1977	BA_1977 polysaccharide deacetylase	1.249
BA_5220	BA_5220 ABC transporter substrate-binding protein	1.244
BA_4751	BA_4751 4-hydroxybenzoyl-CoA thioesterase	1.241
BA_4062	rpmF 50S ribosomal protein L32	1.239
BA_0855	BA_0855 amino acid ABC transporter amino acid-binding protein	1.239
BA_1595	BA_1595 hypothetical protein	1.239
BA_1191	BA_1191 oligopeptide ABC transporter substrate-binding protein	1.237
BA_2534	BA_2534 acetyltransferase	1.236
BA_4448	yqhJ glycine dehydrogenase subunit 1	1.236
BA_2436	asd-1 aspartate-semialdehyde dehydrogenase	1.236
BA_5617	BA_5617 agmatinase	1.228
BA_0010	BA_0010 pyridoxal biosynthesis lyase PdxS	1.228

BA_5222	BA_5222 ABC transporter ATP-binding protein	1.226
BA_4251	mtnA methylthioribose-1-phosphate isomerase	1.225
BA_4038	BA_4038 ylmF protein	1.225
BA_4766	BA_4766 iron compound ABC transporter substrate-binding protein	1.224
GBAA_pXO1_0190	GBAA_pXO1_0190 hypothetical protein	1.221
BA_5047	luxS S-ribosylhomocysteinase 4	1.221
BA_1982	BA_1982 siderophore biosynthesis protein	1.219
BA_1154	rocD ornithine--oxo-acid transaminase	1.219
BA_4818	rpmI 50S ribosomal protein L35	1.218
BA_1983	BA_1983 acyl-CoA synthetas	1.218
BA_4449	gcvT glycine cleavage system aminomethyltransferase T	1.216
BA_0615	BA_0615 iron compound ABC transporter substrate-binding protein	1.214
BA_2258	BA_2258 lipoprotein	1.213
BA_2249	BA_2249 lipoprotein	1.211
BA_3645	BA_3645 oligopeptide ABC transporter substrate-binding protein	1.207
BA_0345	ahpC alkyl hydroperoxide reductase subunit C	1.205
BA_0034	abrB transition state transcriptional regulator AbrB	1.204
BA_0675	BA_0675 zinc-containing alcohol dehydrogenase	1.202
BA_0493	BA_0493 acetylornithine deacetylase	1.201
BA_4043	sigE sporulation sigma factor SigE	0.209
BA_0061	spoIIE stage II sporulation protein E	0.23
BA_3383	BA_3383 lipoprotein	0.263
BA_1636	BA_1636 aminotransferase	0.279
BA_2287	racA polar chromosome segregation protein	0.282
BA_3609	dhaS aldehyde dehydrogenase	0.328
BA_4295	spoIIAB anti-sigma F factor	0.352
BA_4457	aroK shikimate kinase	0.385
BA_2457	BA_2457 O-methyltransferase	0.386
BA_4296	spoIIAA anti-sigma F factor antagonist	0.389
BA_4240	BA_4240 acetyl-CoA acetyltransferase	0.432
BA_4294	sigF sporulation sigma factor SigF	0.438
BA_1290	BA_1290 spore coat-associated protein	0.44

BA_1288	BA_1288 spore coat-associated protein	0.478
BA_1931	BA_1931 branched-chain amino acid ABC transporter branched chain amino acid-binding protein	0.495
BA_4346	cpdB bifunctional 2',3'-cyclic nucleotide 2'-phosphodiesterase/3'-nucleotidase protein	0.502
BA_4491	BA_4491 nucleotidyl transferase	0.521
BA_0372	BA_0372 PTS system transporter subunit IIBC	0.535
GBAA_pXO1_0202	GBAA_pXO1_0202 hypothetical protein	0.541
BA_2071	BA_2071 mutT/nudix family protein	0.558
BA_0368	BA_0368 amino acid ABC transporter ATP-binding protein	0.567
BA_2169	BA_2169 TetR family transcriptional regulator	0.574
BA_2399	BA_2399 metallo-beta-lactamase	0.574
BA_3076	lysP lysine-specific permease	0.574
BA_2632	BA_2632 cytochrome P450 family protein	0.574
BA_2552	BA_2552 carboxyl transferase domain-containing protein	0.579
BA_2109	BA_2109 DEAD/DEAH box helicase	0.582
BA_1817	BA_1817 N-acetylmuramoyl-L-alanine amidase	0.583
BA_5597	BA_5597 DNA-binding response regulator	0.585
BA_0311	BA_0311 isochorismatase	0.585
BA_4149	amiF formamidase	0.587
BA_2352	BA_2352 acyl-CoA dehydrogenase	0.592
BA_1933	BA_1933 branched-chain amino acid ABC transporter ATP-binding protein	0.594
BA_3911	BA_3911 dipeptidase	0.596
BA_2551	BA_2551 enoyl-CoA hydratase	0.596
BA_5606	BA_5606 aminopeptidase	0.601
BA_2553	BA_2553 acetoacetyl-CoA synthase	0.605
BA_2001	BA_2001 intracellular serine protease	0.611
BA_2561	BA_2561 DNA-binding response regulator	0.615
BA_3584	BA_3584 collagenase	0.615
BA_3737	BA_3737 N-acetylmuramoyl-L-alanine amidase	0.618
BA_0898	BA_0898 N-acetylmuramoyl-L-alanine amidase	0.619
BA_5568	BA_5568 sua5/yciO/yrdC/ywlC family protein	0.619
BA_3330	BA_3330 beta-lactam antibiotic acylase	0.621

BA_5248	BA_5248 acetyl-CoA acetyltransferase	0.637
GBAA_pXO1_0079	GBAA_pXO1_0079 surface layer protein	0.638
BA_2872	BA_2872 degV family protein	0.638
BA_4983	BA_4983 homoserine O-acetyltransferase	0.639
BA_0902	BA_0902 ornithine cyclodeaminase	0.639
BA_1228	BA_1228 glucose-1-phosphate thymidylyltransferase	0.64
BA_2379	BA_2379 DinB family DNA polymerase	0.64
BA_2550	mvaB hydroxymethylglutaryl-CoA lyase	0.64
BA_0477	BA_0477 prophage LambdaBa04, tape measure protein	0.644
BA_3368	BA_3368 ribonuclease activity regulator protein RraA	0.645
BA_1130	BA_1130 S-layer protein	0.645
BA_2548	BA_2548 acetyl-CoA carboxylase biotin carboxylase subunit	0.645
BA_0701	qoxC quinol oxidase subunit III	0.646
BA_4558	nadD nicotinic acid mononucleotide adenylyltransferase	0.65
BA_0876	BA_0876 long-chain-fatty-acid--CoA ligase	0.657
BA_0564	BA_0564 ankyrin repeat-containing protein	0.659
BA_2183	BA_2183 neutral metalloprotease	0.659
BA_4671	BA_4671 DNA-binding response regulator	0.66
BA_2814	BA_2814 uvrD/Rep helicase	0.66
BA_1132	aceA isocitrate lyase	0.662
BA_2227	BA_2227 acetyltransferase	0.662
BA_1747	BA_1747 alpha/beta fold family hydrolase	0.662
BA_4561	aroE shikimate 5-dehydrogenase	0.664
BA_0318	BA_0318 MarR family transcriptional regulator	0.666
BA_3578	BA_3578 methyltransferase	0.668
BA_1287	sipW signal peptidase I	0.67
BA_1296	ywdH aldehyde dehydrogenase	0.671
BA_0371	BA_0371 glycosyl hydrolase	0.671
BA_5119	glgP glycogen phosphorylase	0.671
BA_1217	BA_1217 bis(5'-nucleosyl)-tetraphosphatase	0.673
BA_4655	BA_4655 ArsR family transcriptional regulator	0.673
BA_1129	BA_1129 S-layer protein	0.674
BA_2154	spoVS-1 stage V sporulation protein S	0.675

BA_5132	yugI general stress protein 13	0.676
BA_2890	BA_2890 UbiE/COQ5 family methlytransferase	0.678
BA_0936	BA_0936 lipoprotein	0.681
BA_4345	nhaC-3 Na+/H+ antiporter NhaC	0.682
BA_2053	BA_2053 cytosolic long-chain acyl-CoA thioester hydrolase family protein	0.683
BA_2064	csaA chaperone CsaA	0.683
BA_5317	BA_5317 methyl-accepting chemotaxis protein	0.684
BA_0887	eag S-layer protein	0.684
BA_4548	holA DNA polymerase III subunit delta	0.686
BA_2883	BA_2883 lipoprotein	0.686
BA_2720	BA_2720 HAD superfamily hydrolase	0.687
BA_5437	BA_5437 BigG family transcription antiterminator	0.689
BA_0664	rbsR ribose operon repressor	0.691
BA_4823	dnaB DNA replication protein DnaB	0.691
BA_1424	hisZ ATP phosphoribosyltransferase regulatory subunit	0.691
BA_0992	sigB RNA polymerase sigma factor SigB	0.692
BA_5719	BA_5719 DHH subfamily 1 protein	0.692
BA_4109	fosB-2 fosfomycin resistance protein FosB	0.694
BA_1463	BA_1463 ribosomal protein L5 domain-containing protein	0.694
BA_1251	trpC Indole-3-glycerol phosphate synthase	0.694
BA_4399	BA_4399 hemolysin A	0.695
BA_4348	BA_4348 MarR family transcriptional regulator	0.698
BA_5258	BA_5258 cell wall surface anchor family protein	0.699
BA_2420	BA_2420 acetyltransferase	0.7
BA_4493	pstB phosphate transporter ATP-binding protein	0.7
BA_4729	BA_4729 solute-binding family 5 protein	0.701
BA_5257	BA_5257 hypothetical protein	0.704
BA_0140	cbiO cobalt transporter ATP-binding protein	0.706
BA_4369	BA_4369 ribosomal-protein-alanine acetyltransferase	0.706
BA_4628	BA_4628 recombination factor protein RarA	0.707
BA_3912	spoVS-2 stage V sporulation protein S	0.707
BA_2627	cypA cytochrome P450	0.707
BA_2705	BA_2705 endo/excinuclease amino terminal	0.709

	domain-containing protein	
BA_4402	ispA geranyltranstransferase	0.715
BA_1327	BA_1327 phaP protein	0.716
BA_3655	BA_3655 Gfo/Idh/MocA family oxidoreductase	0.719
BA_1635	BA_1635 sodium/solute symporter family protein	0.719
BA_0923	BA_0923 sensor histidine kinase	0.72
BA_0697	BA_0697 acetyltransferase	0.721
BA_4249	BA_4249 3-hydroxybutyrate dehydrogenase	0.721
BA_4237	BA_4237 lipoprotein	0.723
BA_2318	BA_2318 DNA-binding protein	0.723
BA_0327	gabD succinate-semialdehyde dehydrogenase	0.726
BA_0592	ald-1 alanine dehydrogenase	0.726
BA_1040	BA_1040 UvrD/Rep family helicase	0.727
BA_3919	BA_3919 ACT domain-containing protein	0.728
BA_0384	BA_0384 ABC transporter ATP-binding protein	0.73
BA_5203	BA_5203 phosphoglycerate mutase	0.73
BA_3984	BA_3984 DNA-binding protein	0.731
BA_0570	BA_0570 serine/threonine phosphatase	0.731
BA_3699	BA_3699 hypothetical protein	0.731
BA_2079	dal-2 alanine racemase	0.731
BA_2016	BA_2016 luciferase	0.733
BA_5651	BA_5651 lipase/acylhydrolase	0.734
BA_1142	addA ATP-dependent nuclease subunit A	0.734
BA_2608	BA_2608 homoserine dehydrogenase	0.742
BA_2296	BA_2296 CoA-transferase subunit beta	0.742
BA_2547	BA_2547 acyl-CoA dehydrogenase	0.742
BA_4008	rpoZ DNA-directed RNA polymerase subunit omega	0.743
BA_0205	BA_0205 molybdopterin biosynthesis protein	0.744
BA_0401	BA_0401 tellurium resistance protein	0.744
BA_0680	BA_0680 FAD-binding oxidoreductase	0.745
BA_4340	bioD dithiobiotin synthetase	0.745
BA_1376	BA_1376 ABC transporter ATP-binding protein	0.745
BA_4639	recJ single-stranded-DNA-specific exonuclease RecJ	0.746
BA_1515	BA_1515 pyridine nucleotide-disulfide oxidoreductase family protein	0.747
BA_5177	phnA alkylphosphonate utilization operon protein	0.748

	PhnA	
BA_1435	BA_1435 CDP-diacylglycerol--serine O-phosphatidyltransferase	0.749
BA_1162	BA_1162 alpha-amylase 1	0.751
BA_3236	BA_3236 D-cysteine desulfhydrase	0.752
BA_0702	qoxB quinol oxidase subunit I	0.753
BA_1941	BA_1941 MarR family transcriptional regulator	0.754
BA_2003	BA_2003 aldo/keto reductase family oxidoreductase	0.755
BA_0394	BA_0394 type I phosphodiesterase/nucleotide pyrophosphatase family protein	0.755
BA_4923	BA_4923 Gfo/Idh/MocA family oxidoreductase	0.756
BA_3707	BA_3707 Oye family NADH-dependent flavin oxidoreductase	0.757
BA_4874	fabG 3-ketoacyl-ACP reductase	0.757
BA_3070	BA_3070 uridine kinase	0.757
BA_3657	parE DNA topoisomerase IV subunit B	0.757
BA_4858	BA_4858 thioesterase	0.757
BA_5496	BA_5496 ABC transporter ATP-binding protein	0.76
BA_5122	glgC glucose-1-phosphate adenylyltransferase	0.76
BA_0901	BA_0901 proline racemase	0.76
BA_0325	gabT 4-aminobutyrate aminotransferase	0.76
BA_0400	BA_0400 tellurium resistance protein	0.761
BA_4229	BA_4229 maltosaccharide ABC transporter maltosaccharide-binding protein	0.762
BA_4006	priA primosome assembly protein PriA	0.763
BA_4542	hemN coproporphyrinogen III oxidase	0.763
BA_4000	BA_4000 serine/threonine protein kinase	0.764
BA_4953	BA_4953 pullulanase	0.765
BA_1309	glcD glycolate oxidase subunit GlcD	0.765
BA_4389	BA_4389 sensory box sigma-54 dependent DNA-binding response regulator	0.766
BA_2072	BA_2072 amidase	0.767
BA_3440	BA_3440 short chain dehydrogenase/reductase family oxidoreductase	0.767
BA_4394	spo0A stage 0 sporulation protein A	0.767
BA_3672	BA_3672 DNA polymerase III subunit epsilon	0.768

BA_4231	malL oligo-1,6-glucosidase	0.768
BA_0080	BA_0080 negative regulator of genetic competence ClpC/MecB89690:92125	0.769
BA_3409	dxr-1 1-deoxy-D-xylulose 5-phosphate reductoisomerase	0.77
BA_1352	BA_1352 MarR family transcriptional regulator	0.77
BA_3703	BA_3703 phospholipase/carboxylesterase	0.77
BA_5064	feoB ferrous iron transport protein B	0.77
BA_3986	smC chromosome segregation protein SMC	0.77
BA_4516	dnaG DNA primase	0.771
BA_4846	accD acetyl-CoA carboxylase subunit beta	0.773
BA_3463	BA_3463 aldo/keto reductase family oxidoreductase 3185456:3186466 forward MW:37129	0.774
BA_1891	BA_1891 deoxyribonucleoside regulator DeoR	0.774
BA_2377	hup-2 DNA-binding protein HU	0.775
BA_5075	BA_5075 bmrU protein	0.776
BA_3996	rpmB 50S ribosomal protein L28	0.777
BA_1426	hisD histidinol dehydrogenase	0.779
GBAA_pXO1_0199	GBAA_pXO1_0199 hypothetical protein	0.779
BA_5717	BA_5717 replicative DNA helicase	0.781
BA_1503	fer ferredoxin	0.781
BA_0774	BA_0774 pyridine nucleotide-disulfide oxidoreductase, class I	0.781
BA_0044	purR pur operon repressor	0.781
BA_3661	BA_3661 DNA-binding response regulator	0.782
BA_1295	BA_1295 immune inhibitor A metalloprotease	0.782
BA_2860	BA_2860 x-prolyl-dipeptidyl aminopeptidase	0.783
BA_3346	BA_3346 6-aminohexanoate-dimer hydrolase	0.783
BA_2057	BA_2057 oxidoreductase	0.784
BA_4136	BA_4136 PDZ domain-containing protein	0.784
BA_2353	garR 2-hydroxy-3-oxopropionate reductase	0.785
BA_0715	BA_0715 phosphate ABC transporter substrate-binding protein	0.786
BA_4191	BA_4191 TrkA family potassium uptake protein	0.787
BA_3343	BA_3343 alpha/beta fold family hydrolase	0.788
BA_5731	BA_5731 stage 0 sporulation protein J	0.788

BA_5121	glgD glycogen biosynthesis protein GlgD	0.789
BA_1385	BA_1385 2-nitropropane dioxygenase	0.789
BA_3992	BA_3992 fatty acid biosynthesis transcriptional regulator	0.79
BA_3991	plsX glycerol-3-phosphate acyltransferase	0.79
BA_4547	rpsT 30S ribosomal protein S20	0.79
BA_2768	BA_2768 thioredoxin reductase	0.791
BA_1213	ppnK inorganic polyphosphate/ATP-NAD kinase	0.791
BA_2952	BA_2952 acetyltransferase	0.791
BA_5184	BA_5184 nucleotidyltransferase domain-containing protein	0.792
BA_5539	nuoD NADH dehydrogenase subunit D	0.792
BA_1948	BA_1948 lipoprotein	0.793
BA_0004	recF recombination protein F	0.794
BA_3105	BA_3105 acyl-CoA dehydrogenase, short-chain specificreverse	0.794
BA_5123	glgB glycogen branching protein	0.794
BA_1964	BA_1964 mutT/nudix family protein	0.795
BA_5585	BA_5585 TetR family transcriptional regulator	0.795
BA_1483	deoD purine nucleoside phosphorylase	0.795
BA_3257	BA_3257 ArsR family transcriptional regulator	0.796
BA_2919	ssuD alkanesulfonate monooxygenase	0.796
BA_4830	mutM formamidopyrimidine-DNA glycosylase	0.796
BA_4208	ppk polyphosphate kinase	0.796
BA_0202	modB molybdenum ABC transporter permease	0.797
BA_0875	BA_0875 coproporphyrinogen III oxidase	0.797
BA_4728	BA_4728 deaminase	0.797
BA_5249	BA_5249 3-hydroxyacyl-CoA dehydrogenase	0.797
BA_3501	BA_3501 lysozyme	0.798
BA_3913	BA_3913 phosphoesterase	0.799
BA_4318	lolS lolS protein	0.799
BA_2932	BA_2932 glutathionylspermidine synthase	0.8
BA_3847	fruB 1-phosphofructokinase	0.8
BA_0536	bcP bacterioferritin comigratory protein	0.8
BA_5246	BA_5246 acyl-CoA dehydrogenase	0.8
BA_5065	BA_5065 FeoA family protein	0.801

GBAA_pXO1_0037	GBAA_pXO1_0037 nucleotidyltransferase domain-containing protein	0.801
BA_2654	BA_2654 thiJ/pfpI family protein	0.802
BA_3111	BA_3111 aminoglycoside phosphotransferase	0.802
GBAA_pXO1_0206	GBAA_pXO1_0206 hypothetical protein	0.802
BA_2549	BA_2549 acetyl-CoA carboxylase biotin carboxyl carrier protein subunit	0.803
BA_3610	fabG 3-oxoacyl-ACP reductase 3319812:3320552 reverse MW:26512	0.803
BA_1558	BA_1558 glycosyl transferase family protein	0.804
BA_0996	BA_0996 sensor histidine kinase/response regulator	0.804
BA_1545	qcrB cytochrome b6	0.804
BA_0079	BA_0079 ATP:guanido phosphotransferase	0.804
BA_2650	BA_2650 penicillin-binding protein	0.805
BA_4562	BA_4562 GTP-binding protein Yqe 4146595:4147701 reverse MW:41283	0.805
BA_4492	BA_4492 phosphate transport system regulatory protein PhoU	0.806
BA_1418	ilvH acetolactate synthase 3 regulatory subunit	0.806
BA_1152	BA_1152 fumarylacetoacetate hydrolase	0.806
BA_5414	BA_5414 carboxyl-terminal protease	0.806
BA_2354	mmsA-1 methylmalonic acid semialdehyde dehydrogenase	0.806
BA_1797	pyrH uridylate kinase	0.807
BA_3993	recG ATP-dependent DNA helicase RecG	0.807
BA_0375	topB-1 DNA topoisomerase III	0.808
BA_0046	BA_0046 endoribonuclease L-PSP	0.808
BA_3842	hfq RNA-binding protein Hfq	0.808
BA_4331	ribD riboflavin biosynthesis protein RibD	0.809
BA_4717	racE-2 glutamate racemase	0.809
BA_4536	BA_4536 16S ribosomal RNA methyltransferase RsmE	0.809
BA_2164	BA_2164 ABC transporter ATP-binding protein	0.809
BA_0014	BA_0014 deoxynucleoside kinase	0.81
BA_3945	rpsO 30S ribosomal protein S15	0.81
BA_1008	BA_1008 DNA repair exonuclease	0.811

BA_2263	BA_2263 mechanosensitive ion channel family protein	0.811
BA_5120	glgA glycogen synthase	0.811
BA_4360	sdhA-2 iron-sulfur-dependent L-serine dehydratase subunit alpha	0.811
BA_5049	BA_5049 carbonic anhydrase	0.811
BA_2596	BA_2596 acetamidase/formamidase	0.812
BA_2111	BA_2111 glyoxalase	0.812
BA_2265	BA_2265 LuxR family DNA-binding response regulator	0.813
BA_5590	BA_5590 ferredoxin, 4Fe-4S	0.813
BA_0084	ispD 2-C-methyl-D-erythritol 4-phosphate cytidylyltransferase	0.814
BA_4308	BA_4308 purine nucleoside phosphorylase	0.814
BA_0095	secE preprotein translocase subunit SecE	0.815
BA_5195	fbp-1 fructose 1,6-bisphosphatase II	0.815
BA_4994	BA_4994 TrkA domain-containing protein	0.815
BA_2818	recQ-2 ATP-dependent DNA helicase RecQ	0.815
BA_0399	BA_0399 tellurium resistance protein	0.815
BA_0599	BA_0599 neutral protease	0.816
BA_4013	BA_4013 fibronectin/fibrinogen-binding protein	0.817
BA_1629	cspB-1 cold shock protein CspB	0.817
BA_2405	BA_2405 hydrolase	0.818
BA_0021	recR recombination protein RecR	0.818
BA_3514	BA_3514 metallo-beta-lactamase	0.818
BA_4024	pyrK dihydroorotate dehydrogenase electron transfer subunit	0.818
BA_1836	BA_1836 polysaccharide deacetylase	0.819
BA_0056	BA_0056 S4 domain-containing protein	0.82
BA_5380	clpP ATP-dependent Clp protease proteolytic subunit	0.82
BA_0410	BA_0410 heavy metal-transporting ATPase	0.821
BA_4777	BA_4777 DNA-binding response regulator	0.821
BA_1758	BA_1758 GntR family transcriptional regulator	0.821
BA_0344	ahpF alkyl hydroperoxide reductase subunit F	0.821
BA_0965	BA_0965 dihydroxyacetone kinase	0.822
BA_0052	mfd transcription-repair coupling factor	0.822

BA_5074	hpt-2 hypoxanthine-guanine phosphoribosyltransferase	0.823
BA_2850	BA_2850 mandelate racemase/muconate lactonizing protein	0.823
BA_3985	ftsY signal recognition particle-docking protein FtsY	0.823
BA_5472	dat-2 D-amino acid aminotransferase	0.823
BA_3905	mutS DNA mismatch repair protein MutS	0.823
BA_4153	ctaD cytochrome c oxidase subunit I	0.824
BA_2048	BA_2048 dedA family protein	0.825
BA_3095	BA_3095 LamB/YcsF family protein	0.825
BA_1537	aroF-1 chorismate synthase	0.825
BA_2135	moeA-1 molybdopterin biosynthesis protein MoeA	0.826
BA_4650	ruvB Holliday junction DNA helicase RuvB	0.826
BA_5639	BA_5639 D-alanyl-D-alanine carboxypeptidase	0.827
BA_4336	bioB biotin synthase	0.827
BA_5334	vacB ribonuclease R	0.827
BA_5079	BA_5079 aldo/keto reductase family oxidoreductase	0.828
BA_4400	dxs 1-deoxy-D-xylulose-5-phosphate synthase	0.828
BA_1250	trpD anthranilate phosphoribosyltransferase	0.828
BA_5714	yycG sensory box histidine kinase YycG	0.829
BA_4276	scpB segregation and condensation protein B	0.829
BA_4622	BA_4622 helicase	0.83
BA_4147	BA_4147 hypothetical protein	0.83
BA_1952	BA_1952 NLP/P60 family protein	0.83
BA_2673	BA_2673 chitosanase	0.83
BA_2360	BA_2360 exonuclease	0.83
BA_0830	BA_0830 AsnC family transcriptional regulato	0.831
BA_5667	BA_5667 response regulator	0.832
BA_4477	murG UDPdiphospho-muramoylpentapeptide beta-N-acetylglucosaminyltransferase	0.832
BA_4815	BA_4815 M42 family peptidase	0.833
BA_3966	codY transcriptional repressor	0.873

Protein extraction and iTRAQ analysis. Proteomic analysis of *B. anthracis* cells was performed according to methods reported previously. Specifically, protein samples cultured in DSM were collected at T_1. Proteomic analysis of *B. anthracis* cells was performed according to methods

reported previously [1]. Protein concentrations were determined using the Bradford method. Solutions containing 100 μg protein were loaded into a 10 KDa ultrafiltration (OD010C35, PALL, Port Washington, NY, USA) tube and digested using trypsin for 12 h at 37°C after an initial reduction step with DTT and alkylation. A16R, Δ*spoVG*, and mix sample were labeled with 113, 114, and 116 mass tags, respectively. Data was analyzed using Proteome Discoverer™ 2.2 software (Thermo, San Jose, CA, USA), and the Ames chromosome amino acids database (NCBI Accession: NC_003997.3) was used. The false-positive rate of peptide identification was controlled below 1%. Protein fold changes of ≥1.2 with at least one unique peptide were considered significantly different.

Table S3. List of differentially-expressed proteins related to growth and spore formation.

Accession	Description	Abundance Ratio	Δ*spoVG*[a]
**Growth metabolic pathway**			
BA_0701	qoxC quinol oxidase subunit III	0.646	down
BA_0702	qoxB quinol oxidase subunit I	0.753	down
BA_4240	acetyl-CoA acetyltransferase	0.432	down
BA_1288	spore coat-associated protein	0.478	down
BA_1290	BA_1290 spore coat-associated protein	0.44	down
BA_3966	codY transcriptional repressor	0.873	down
**Sporulation Process**			
BA_0061	spoIIE stage II sporulation protein E	0.23	down
BA_4295	spoIIAB anti-sigma F factor	0.352	down
BA_4296	spoIIAA anti-sigma F factor antagonist	0.389	down
BA_4294	sigF sporulation sigma factor SigF	0.438	down
BA_4394	spo0A stage 0 sporulation protein A	0.767	down
BA_0093	sigH RNA polymerase factor sigma-70	1.9	up
BA_5581	spo0F stage 0 sporulation protein F	1.285	up

[a]: up and down denote the upregulated and downregulated proteins of the *spoVG* mutant strain with respect to the wild-type *B. anthracis* A16R strain

Figure S1

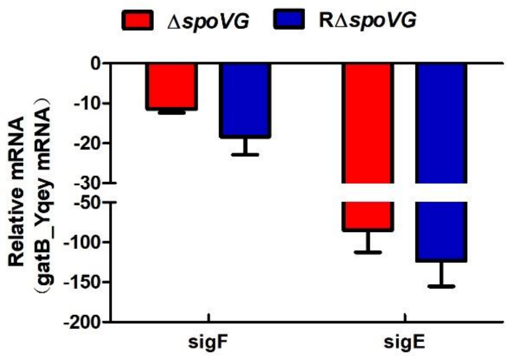

Figure S1. Verification of differentially expressed genes using RT-qPCR. Relative expression of *sigF* and *sigE* genes was determined in the Δ*spoVG* and RΔ*spoVG* strains compared with A16R at T_1. The mRNAs of both *sigF* and *sigE* were significantly down-regulated in Δ*spoVG* compared with A16R. Values are mean ± SD of triplicate experiments.

Table S4. Conservation of comC between *B. anthracis* and *B. subtilis*.*

Query	Sbjct	Range	Identities
comC (*B. anthracis)*<br><br>240bp	comC (*B. subtilis)*<br><br>248bp	9-229	39%

* Sequence alignment results are shown below

Query 11

GMVFGSFFMLIAMRIPLGESIIIPRSHCHYCKYVLKPKELIPIISFCIQRGRCTNCKRKI 70

G++ GSF+ RIPL SII PRS C +C+ L P ELIPI+SF Q+G+C +C +I

Sbjct 9 GLILGSFYYTAGCRIPLHLSIIAPRSSCPFCRRTLTPAELIPILSFLFQKGKCKSCGHRI

68

Query 71 SILYVIFELVTGIICLLTVYMIGVERELIIILSLFSLLLIISVTDYIYMLIPNRILAWFS

130

S +Y ELVT + G+ EL + SLL+I++VTD +MLIPNRIL +F

Sbjct 69 SFMYPAAELVTACLFAAAGIRFGISLELFPAVVFISLLIIVAVTDIHFMLIPNRILIFFL

128

Query 131

CLLILECVFVPLVTWTESIVGSGVIFILLYCMQKIYPEGLGGGDIKLLSLLGFIAGLKGV 190

L + PL +W ++G+ F+ L + I G+GGGDIKL +++GF+ G+K +

Sbjct 129

PFLAAARLISPLDSWYAGLLGAAAGFLFLAVIAAITHGGVGGGDIKLFAVIGFVLGVKML 188

Query 191 FMILFLSSFFSLCFFGAGLVLKRMKMRTQIPFGPFISLGAI 231

F S + A ++ R+ R +PF P I+ G+I

Sbjct 189 AAAFFFSVLIGALYGAAAVLTGRLAKRQPLPFAPAIAAGSI 229

Figure S2

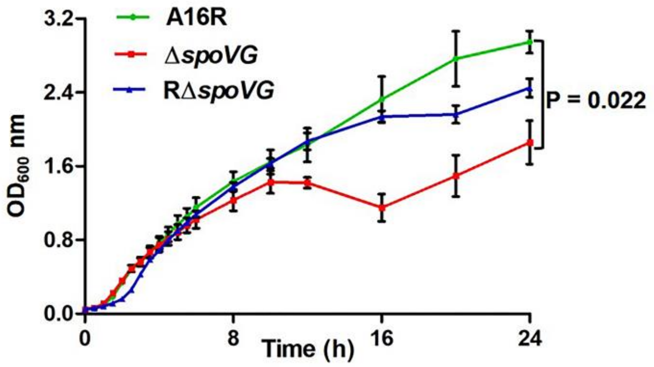

Figure S2. Effect of SpoVG on growth. Growth curve assays were performed in DSM (A16R, green; Δ*spoVG*, red; RΔ*spoVG*, blue; repeated measures ANOVA, F = 348, P = 0.000).

REFERENCES

1. Han, J., P. Gao, S. Zhao, X. Bie, Z. Lu, C. Zhang, and F. Lv. "Itraq-Based Proteomic Analysis of Li-F Type Peptides Produced by *Paenibacillus polymyxa* Jsa-9 Mode of Action against *Bacillus cereus*." Journal of Proteomics 150 (2017): 130-40.
